# Machine learning approaches for large scale classification of produce

**DOI:** 10.1038/s41598-018-23394-3

**Published:** 2018-03-27

**Authors:** Otkrist Gupta, Anshuman J. Das, Joshua Hellerstein, Ramesh Raskar

**Affiliations:** Massachusetts Institute of Technology, Cambridge, MA 02139 USA

## Abstract

The analysis and identification of different attributes of produce such as taxonomy, vendor, and organic nature is vital to verifying product authenticity in a distribution network. Though a variety of analysis techniques have been studied in the past, we present a novel data-centric approach to classifying produce attributes. We employed visible and near infrared (NIR) spectroscopy on over 75,000 samples across several fruit and vegetable varieties. This yielded 0.90–0.98 and 0.98–0.99 classification accuracies for taxonomy and farmer classes, respectively. The most significant factors in the visible spectrum were variations in the produce color due to chlorophyll and anthocyanins. In the infrared spectrum, we observed that the varying water and sugar content levels were critical to obtaining high classification accuracies. High quality spectral data along with an optimal tuning of hyperparameters in the support vector machine (SVM) was also key to achieving high classification accuracies. In addition to demonstrating exceptional accuracies on test data, we explored insights behind the classifications, and identified the highest performing approaches using cross validation. We presented data collection guidelines, experimental design parameters, and machine learning optimization parameters for the replication of studies involving large sample sizes.

## Introduction

The combination of optical spectroscopy, image analysis, chemometric, and data-centric methods has been shown to be an attractive approach for a variety of applications including sorting^1,2^ and determination of produce quality^3–5^. These methods have been used to detect markers which relate to the ripening^6,7^, damage^8^, and spoilage^9^ of produce. More recently, image analysis has also been used to complement spectral information due to the availability of large datasets. Typically, machine learning approaches for produce classification use a combination of linear discriminant analysis (LDA), principal component analysis (PCA), and SVMs with a kernel function^10,11^. El-Bendary *et al.* investigated tomato ripeness with a SVM on 250 visible-spectrum photos, and achieved an accuracy of 90.8%^12^. Another work by Elhariri presented an image classification system that determined tomato ripeness using 175 visible-spectrum images, and achieved an accuracy of 92.72% with a SVM^13^. There has also been investigation of age or geographic origin estimation using similar methods. An accuracy of 98% for classifying 166 samples of persimmon fruit into 7 different regions was accomplished by Khanmohammadi *et al.* using Fourier transform near infrared (FT-NIR) spectrometry. Additionally, Schmutzler *et al.* showed that non-invasive surface scanning near-infrared reflectance spectroscopy (NIRS) could be used to distinguish 160 apples grown in South Tyrol, Italy from 235 apples grown in 20 other countries^14^. Dan *et al.* achieved 96.7% accuracy in classifying 1500 oranges into 15 regions in China using a variety of machine learning approaches such as decision trees, K nearest neighbors (KNN), Naive Bayesian, SVM, and an artificial neural network (ANN). They showed that extracting the juice from the sample and the decision tree were instrumental in achieving high classification accuracies^15^.

More recently, there has been research on quantitatively distinguishing between organic and non-organic produce. A study by Hohmann *et al.* used Hydrogen nuclear magnetic resonance (H-NMR) and invasive chemometric techniques to determine a significant difference between organic and non-organic classes^16^. Further, Laursen *et al.* utilized chemometrics and spectroscopy to attempt to classify organic vs. non-organic fruit production, but found that the diversity of the fertilization practices challenged chemometric methods^17^. However, analysis of biomarkers in lettuce by Flores *et al.* demonstrated successful classification of organic vs. non-organic fruits with an accuracy of 90.4%^18^.

Most of the aforementioned examples, though effective, employed invasive methods like Brix or other chemometric analyses to obtain the data needed for machine learning analysis, effectively eliminating the possibility of large datasets. This becomes a major drawback because small sample sizes, typically in the range of several hundreds, lead to poor classification accuracies when machine learning methods are applied. More attractive methods eschew invasive approaches while arriving at comparable results using optical spectroscopy and machine learning techniques, requiring minimal sample preparation and sorting or classification that can be done instantaneously. These non-invasive methods have been shown to be effective in distinguishing organic from non-organic produce, locating geographic origin, and testing for produce spoilage. NIRS and forms of discriminant analysis and genetic algorithms were utilized to classify the growing methods of asparagus with up to 91% accuracy by Sanchez *et al*.^19^ and lettuce with up to 95.4% accuracy by Brito *et al*.^20^ respectively.

In this study, we propose an approach for large dataset collection (up to 2 orders of magnitude larger than related works) and classification of several types of produce in the assembly line sorting stage. Sample sizes for produce in the range of 500–14000 were each scanned for several types of produce including apples, strawberries, tomatoes, grapes, oranges, mushrooms, onions and potatoes. These large sizes open up possibilities of employing different data centric approaches, and also provide realistic scenarios of applications. We used SVM approaches to perform sorting^21^ based on taxonomy and obtained accuracies in the range of 96–99% across different types and varieties of produce. Furthermore, we performed vendor (farmer) classification using visible and NIR spectroscopy and achieved near 100% classification of farmer classes for the same variety of produce. Using cross validation techniques, we identified the best hyperparameters and kernel functions when deciding which SVM to use over the test set. We also presented guidelines and discussed challenges of performing studies involving large datasets which should benefit future explorations in this field.

## Results

**Figure 1:**
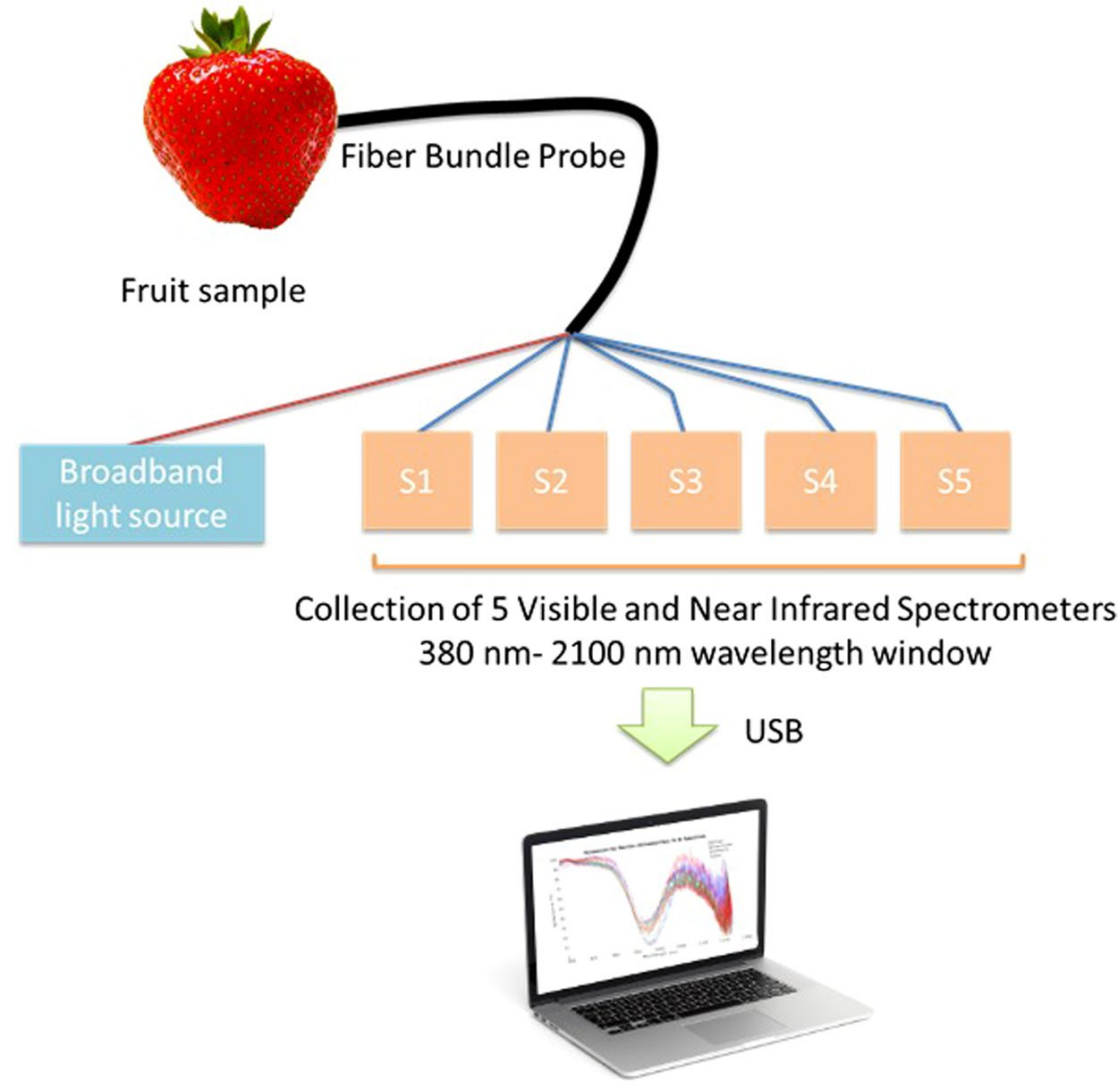
Schematic of the data collection setup. A broadband source was used to illuminate the sample and the reflected signal was collected using an optical fiber probe that served as an input to 5 UV-VIS-NIR spectrometers.

The data collection setup utilized a tungsten light source probe housed in an optical fiber reflection probe. Several optical collection fibers were inputs to 5 spectrometers, which had different bandwidths ranging from 400–2100 nm. The setup was installed at several sorting facilities which allowed quick scanning and large volume data collection (Fig. 1). The obtained data was scrutinized to make sure that the signal-to-noise ratio (SNR) was appreciable, and that the characteristic reflection and absorption bands were in agreement with existing literature.

**Figure 2:**
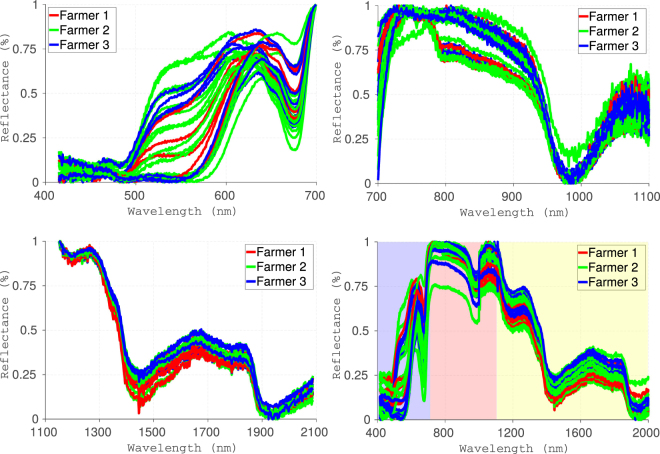
Hyperspectral responses for Fuji apples in the 400–700 nm (top left), 700–1100 nm (top right), 1100–2000 nm (bottom left), and combined spectrum ranges (bottom right). Measurements in combined spectrum (400–2000 nm), includes visible (denoted in blue background), NIR 1 (red background) and NIR 2 (yellow background).

In the case of Fuji apples, we observed a characteristic broad green-red reflection peak in the 500–650 nm range due to the presence of anthocyanins–the pigments responsible for the apples’ color^22^, as shown in Fig. 2. We also observed the reflection dip at around 680 nm which is characteristic of chlorophyll absorption in the skin^22^, as shown in Fig. 2. These visible spectral features were consistent over all the types of produce scanned. Next, we observed a reflection dip or a local absorption maximum in the 900–1000 nm band, as shown in Fig. 2. This band is attributed to the C-H stretching third overtone and linked to the sugar content in the sample^23^. In the NIR portion of the spectrum (1100–2100 nm), we observed the moisture bands at 1440 nm and 1920 nm, as shown in (Fig. 2). This is a set of characteristic water absorption bands that is generally seen in many biological materials^24^. Hence it was ascertained that the measurements made by the probe were accurate and were in agreement with existing reports. This information provided critical insights into the type of produce and its properties like ripeness, moisture content, and anthocyanin content. These parameters assisted in the classification of produce using several labels as presented in the next section.

### Taxonomy classification

The spectral data was pre-processed by discarding the first and last points of every measurement due to low SNR and normalizing the rest between 0 and 1. Classification was performed using 4 spectral regions of the reflectance i.e visible (400–700 nm), NIR 1 (700–1100 nm), NIR 2 (1100–2000 nm) and the composite spectra (400–2100 nm) (see Fig. 2). In the visible band, we observed classification accuracies in the range of 0.828–0.99 for different types of produce for sample sizes of 900 and above. This is expected as the produce had distinct color appearances which are easily classified, as shown in Table 1. We observed lower classification accuracies for strawberries and apples, as some darker apple varieties could have similar color characteristics as strawberries. Overall, lower classification accuracies were observed in the NIR 1 band, mostly in the 0.840–0.99 range; this can be attributed to some visible features around 700 nm and the sugar content in the 900–1000 nm band as shown in Table 1. For the NIR 2 band, we observed lower classification accuracies compared to the visible because only the moisture content is captured in this band, as shown in Table 1. The accuracies range from 0.836–0.987, as the samples had dissimilar water content. Finally, the composite band had accuracies in between those of the visible, NIR 1, and NIR 2 bands. However, the 0.902–0.981 range is promising for the dataset in the case of the composite band. In all the cases, we separated data into 30–70 test-train split and used the training data to select and train classifiers. We relied on libsvm^25^ to train maximum margin classifiers, using cross validation methods to select the best hyperparameters, and we validated the trained model on the separated test data to report final results. Confusion matrix for fine grained classification is made available in supplementary material.

**Table 1:** Classification accuracies for fine grained taxonomy of fruits and vegetables.

Fruit Type	Number of Classes	Number of Samples	Visible	NIR 1	NIR 2	Composite
(Organic/Inorganic)			(0–700 nm)	(700–1100 nm)	(1100–2000 nm)	(0–2000 nm)
Apples	8	13808	0.915	**0**.**943**	0.836	0.906
Strawberries	2	980	0.828	0.84	0.917	**0**.**942**
Grapes	2	947	**0**.**973**	0.906	0.867	0.921
Oranges	4	2599	0.94	0.911	**0**.**987**	0.981
Mushrooms	3	1217	**0**.**99**	**0**.**99**	0.941	0.943
Onions	2	2686	**0**.**99**	**0**.**99**	0.892	0.903
Bell Peppers	5	1483	**0**.**975**	0.959	0.954	0.945
Jalapeno Chilli	3	3292	0.964	0.9	**0**.**979**	0.976
Potatoes	3	5541	**0**.**981**	0.949	0.962	0.963
Tomatoes	6	3718	**0**.**945**	0.906	0.876	0.902

### Farmer classification

Results were also able identify the source of origin of produce with very high accuracy when farmer label was used for classification. Since different farmers at different geographical locations could cultivate different varieties of produce, we first grouped the data by the specific varieties and trained an SVM on that variety. We used features from visible, NIR 1, NIR 2, and composite features to train SVM similar to the taxonomy classification. Sample sizes in this study were in the range of 500–1700 for the same sub-variety of produce. Accuracies of 0.962 and above were observed for every produce type across the 4 bands as summarized in Table 2. This result is also presented as a confusion matrix for the case of Gala and Fuji apple varieties as shown in Table 4. We observed that the random assignment for Gala apples is 68% as compared to the classification accuracy of 99%. For the case of Fuji apples, we observed a random assignment of 59% as compared to 99% classification. We obtained a very high accuracy in each individual class, demonstrating the robustness of our method even in presence of unbalanced data (see Table 4).

**Table 2:** Farmer classification accuracies from various spectra using linear SVMs.

Fruit Type	Number of Classes	Number of Samples	Visible	NIR 1	NIR 2	Composite
(Organic/Inorganic)			(0–700 nm)	(700–1100 nm)	(1100–2000 nm)	(0–2000 nm)
Fuji Apples	3	1683	0.962	**0**.**992**	0.981	0.982
Gala Apples	3	753	0.987	0.991	**0**.**992**	**0**.**99**
Halo Oranges	2	725	**0**.**99**	**0**.**99**	**0**.**99**	**0**.**99**
Red Bell Peppers	3	510	**0**.**99**	0.98	**0**.**99**	0.994
Red Potatoes	3	741	0.988	0.986	**0**.**99**	**0**.**99**
Russet Potatoes	2	1140	0.994	**0**.**99**	**0**.**99**	**0**.**99**
Steak Tomatoes	3	690	0.981	**0**.**99**	0.991	0.985

**Table 4:** Confusion matrix for farmer classification over gala (left) and Fuji (right) apples. For gala apples our net accuracy is 99% compared to 68% for random assignment. For Fuji apples net accuracy is 99% compared to 59% for random assignment.

	farmer 1	farmer 2	farmer 3		farmer 1	farmer 2	farmer 3
farmer 1	0.99	0.00	0.01	farmer 1	0.96	0.03	0.01
farmer 2	0.03	0.97	0.00	farmer 2	0.00	0.99	0.01
farmer 3	0.00	0.00	1.00	farmer 3	0.00	0.00	1.00

### Organic vs. non-organic classification

Additionally, we were able to use spectral information to separate *organic* fruit samples from rest of samples. We trained and tested over fine grained taxonomy to ensure that the classifier learned independently from produce sub-type. Linear SVMs performed the best, and we observed that visible, infrared, or both components can perform well depending on the sub-species being classified. We obtained accuracies between 96% and 99% when performing classification between organic and non-organic produce (see Table 3). Though we expected the presence of nitrogen-15 (often found in organic fertilizer) to be important in distinguishing a fruit’s organic nature, the high performance on all 4 spectral ranges indicates otherwise.

**Table 3:** Classification accuracy when identifying organic vs non-organic fruit.

Fruit Type	Number of Classes	Number of Samples	Visible	NIR 1	NIR 2	Composite
(Organic/Inorganic)			(0–700 nm)	(700–1100 nm)	(1100–2000 nm)	(0–2000 nm)
Gala Apples	2	3358	0.966	0.874	**0**.**988**	0.982
Red Delicious Apples	2	1095	**0**.**988**	0.938	0.975	0.984
Naval Oranges	2	423	0.97	0.956	**0**.**971**	0.968
Green Onions	2	316	0.904	**0**.**99**	0.979	0.989
Green Bell Peppers	2	119	0.919	0.969	0.906	**0**.**99**

## Discussion

A combination of non-invasive methods like spectroscopy and data-centric approaches can be effective in a range of applications from sorting to distinguishing organic produce to tracing the farm. Small changes in the color, sugar, and moisture content are key factors that contribute to the high classification accuracy. Data collection has been a bottle neck in the past, and most studies have reported datasets in the range of several hundreds. The current study overcomes this barrier and also implements machine learning for several types of produce. This was feasible due to collaborative efforts with institutions that typically deal with very large amounts of produce, e.g. supermarkets. Additionally, this was made feasible by the availability of compact spectrometers and the ease of data manipulation.

High classification accuracies were obtained because of the large sample sizes which mitigated sample-to-sample fluctuations. These accuracies can also be attributed to better instrumentation and data collected with good SNR. Instrumentation that not only has high spectral resolution but also a good analog to digital converter bit depth (16 bit and above) was responsible for accurate results. We were able to reject the noisy portions of the spectra typically present at the lower and higher edges of the spectrum, leading to significant enhancement the classification accuracy.

Overfitting remains an issue of major concern when applying machine learning to new application areas. In *overfitting*, the classifier learns features specific to the dataset, leading to low test accuracy and poor generalization when used in real world scenarios. Previous works relied on much smaller datasets making them far more susceptible to overfitting leading to poor generalization of the classifier. We tackle the issue of overfitting by relying on a large sized dataset and maintaining test set integrity when training support vector machine classifiers^26^. In all of our experiments, we first isolated 30% of the produce samples for testing, keeping the remaining dataset for training and hyperparameter selection^27^. We selected optimal hyperparameters by using 4 fold cross validation while training. This included splitting training data further into a 25% validation set and comparing results from multiple choices of hyperparameters while performing a grid search over hyperparameter space. Hyperparameters which yielded the best cross validation accuracy were used to train a model over entire training data. Finally, the trained model was used to provide classification results over the test set isolated in the first step. This methodology ensured that the test set was only used for inference, did not influence the model training procedure in any way, and provided the best possible results when selecting hyperparameters.

Our experiments reveal that linear classifiers performed the best when attempting classification over fine grained taxonomy and farmers. While polynomial kernels performed the worst, both linear and radial basis kernels performed well, with linear kernels winning with small margins. We think this is because linear methods provided the simplest model with fewer parameters and polynomial kernels may have a tendency to overfit^28^, leading to overall poor performance over the test set. A welcome consequence of using linear classifiers is that it makes the entire classification pipeline simpler and more computationally feasible, providing easier implementation in portable and embedded applications, paving the way for consumer devices. We observed that combining features from both visible and infrared spectrum can yield higher accuracy for produce like strawberries. While more data is always welcome in machine learning, more features may also reduce accuracy by increasing confusion and adding inter class noise. More features also require more parameters and a larger model, making them susceptible to overfitting, leading to poor generalization performance as observed when classifying apples or grapes.

In our experiments, we discovered significant accuracy improvements by correctly selecting hyperparameters and kernels. For example, an accuracy of only 71% was observed when identifying apples with a fixed set of hyperparameters. Similarly, an accuracy of 42% was observed when using *polynomial* kernels instead of *linear* or *rbf* kernels, leading to the conclusion that both kernel and hyperparameter selection are important to our method. While we pooled samples from various sources when performing taxonomic classification, fixing sources of origin greatly improved accuracy from 88% to 97–98%. This is expected since produce samples from the same origin source should have reduced inter class variation, leading to better classification accuracy. Similarly, higher accuracies were also seen when training without normalization, which may have resulted from extra information about color tones or scaling biases introduced during measurement.

There are several aspects of the study that can lead to classification failure or difficulty in interpretation of data. First, the user collecting the data needs to perform the scan in a homogeneous manner by choosing a similar scan location for the same type of produce. They also need to have a systematic, error-free method to enter any produce notes (e.g. misspelled names of farmers could generate a second, unnecessary label). In this case, data was also collected with options for user inputs that could capture abnormalities in the produce, e.g. visible spoilage or damage. This helps in the detecting outliers that may or may not be selected as a part of the test dataset. However, this can be a source of subjectivity and lead to errors. As is the case with large data collection exercises, care needs to taken to capture the metadata accurately so that the analysis process is simplified. Second, the information about the harvest time is critical information that needs to be collected. It is possible that in our farmer classification results, the produce was at different stages of maturity and that had a significant contribution to the overall accuracy. More controlled studies are needed to understand this further.

Both the hyperparameter and the kernel can dramatically influence the performance of SVM and a grid search is required to produce the optimal model. As a comparison, we have included the model selection graphs in our supplementary material. Another factor that influences accuracies is the systematic noise present in the beginning and end of hyperspectral measurements. We remove this error by clipping the signal at both ends and normalizing it. Resolution of each instrument can affect accuracy greatly, as observed in Tables 1 through 4 of supplementary material. We envision more such studies that utilize large datasets and the application of other novel classification methods like deep neural networks to increase the accuracy^29,30^ or work on smaller datasets if possible. This will not only help in sorting or distribution at a vendor level, but also may work at a consumer level to improve the trust and transparency around food products.

## Methods

### Spectrometers

A set of 5 UV-VIS and NIR spectrometers were used for spectral data acquisition. Three UV-VIS spectrometers SPARK-VIS (Ocean Optics) FLAME-S (Ocean Optics) and QEPRO (Ocean Optics) were used to gather data in the visible spectrum. SPARK-VIS had a spectral range of 380–700 nm, FLAME-S had a range of 350–1000 nm and the QEPRO operated in the 400–1150 nm band. The QEPRO had superior performance characteristics as compared to SPARK-VIS and FLAME-s with respect to the signal to noise ratio. Two NIR spectrometers were used in the study with varying wavelength ranges. FLAME-NIR (Ocean Optics) and NIRQUEST (Ocean Optics) spectrometers had wavelength ranges from 950–1650 nm and 900–2100 nm, respectively. The NIRQUEST had a superior performance and a better signal to noise ratio. Spectrometers used in this study had a very good signal to noise ratio and low dark counts. We validated all the spectra obtained from various produce groups with previously published results and found a good match with all the spectral features. The justification of using several spectrometers was to evaluate the performance in a field setting so that the best data could be selected for analysis.

### Spectrometer Integration and probe design

The optical input of all the spectrometers used in the study were combined using an optical fiber reflectance probe. A broadband halogen light source was coupled with the reflectance probe for the purpose of illumination. The probe optical fiber bundle consisted of 25 collection fibers (5 for each spectrometer) and 1 illumination fiber. The output of the spectrometers was connected to a USB hub which was in turn connected to a laptop to capture and store data.

### Data Collection

For every spectral sample collected, the corresponding metadata was captured using a graphic user interface. The following information was entered into the metadata file: Sample information, taxonomy, vendor name, location and geo-location of vendor, supplier information and the harvest date. Other instrument parameters were also recorded into the metadata. There were options for the user to enter other notes like visible spoilage etc. in the metadata information. 7491 strawberries, 5875 apples (different varieties), 1863, tomatoes, 1150 potatoes, 864 oranges, 381 pears, 830 leafy greens, 372 bell peppers and 271 grapefruits were considered for the study. It is to be noted that there were 5 spectral samples obtained for each sample scanned.

### Reflectance and transmittance calculations

The data obtained from the spectrometer was converted to an absorbance and reflectance spectrum for both visible and infrared bands of the measurement. For consistency in the visible and NIR spectral analysis, the reflectance spectrum was chosen under the assumption there was no transmission through the sample. This assumption is valid as there was no sample preparation involved, and the produce sample had a very large optical path length. For the data analysis, both edges of the spectrum were trimmed as the noise was high in these regions.

### Data Analysis

The data for a single produce sample comprised of 5 spectral signals from the several spectrometers used in the study. Each of the 5 signals had both a reflection and transmittance spectrum. As mentioned earlier, all samples were opaque, and thus a reflectance or transmittance spectrum was chosen as the input to the machine learning algorithm. Each reflectance or transmittance spectrum was divided into visible component, NIR component and composite spectrum categories to ascertain the contributions of each spectral component in the overall classification of the produce. Specifically, the visible (400–700 nm), NIR 1 (700–1100 nm), NIR 2 (1100–2000 nm) and composite (400–2000 nm) bands were used in the analyses.

### Machine learning implementation: SVMs

We modeled the tasks of identifying farmers and taxonomy as a *multi-class* classification problem and relied on the use of SVMs^31^ to perform instance classification. SVMs constitute a class of supervised learning models for performing classification over single and multiple classes^32^. Modern SVM algorithms rely on constructing optimal hyperplanes (or maximum margin classifiers^33^) in higher dimensional spaces thereby dividing input data into multiple-classes. Higher margin in classifier can lower the generalization error by increasing the distance between hyperplane and class boundaries. Similarly pre-projecting data in higher dimensional spaces (*polynomial*, *rbf* kernels) can reduce misclassifications if the data is not linearly separable^34^.

In our experiments, we tried linear, polynomial and rbf kernels and found that linear kernels performed the best. We only applied minor preprocessing on data, which involved removing the datapoints at extreme ends of spectrum and re-normalizing the data between 0 and 1. When selecting the best SVM model, we varied 3 different hyperparameters - hyperparmeter *ν* which controls fraction of support vectors selected, *t* denotes the kernel which can be RBF, Linear or Polynomial, and *η* controls number of training set partitions for cross validation purposes. For optimal hyperparameter selection, we performed a grid search^35^ over the hyperparameter *ν*^36^ between 0.1 and 1 and selected the best possible value using 4 fold cross validation^37^ over the training set. The kernel itself was controlled by varying the hyperparameter *t* which could be 0, 1 or 2 for linear, polynomial and rbf kernels. Cross validation parameter *η* was set to 4 for four fold cross validation. We then used these hyperparameters to train a SVM over the entire training set and reported accuracies as seen on the test set. This procedure ensured that hyperparameters were completely based on training set itself.

All experiments were run on an 8 core Intel Xeon^38^ server equipped with 16 gigabytes of memory, 256 gigabytes of flash storage and Nvidia Quadro graphics card containing 1 gigabyte of VRAM. Hyperparameter selection required multiple iterations of train and cross validation loops and took several hours when training on a large number of samples. The trained model, requiring only a few megabytes, is easily deployable on smaller devices for test and inference purposes. However, actual training and analysis of data would require a slightly more powerful hardware setup like the one used during the research performed in this paper. The dataset was stored and formatted in JSON format and models were trained using *libsvm* bindings for MATLAB.

## Electronic supplementary material

Supplementary Material
