# Supplementary material for: Machine learning approaches for large scale classification of produce
Source: Sci Rep. 2018 Mar 27;8:5226. doi: 10.1038/s41598-018-23394-3 (PMC5869718; doi:10.1038/s41598-018-23394-3)
Supplement: Supplementary file 1 — Supplementary Material [file 41598_2018_23394_MOESM1_ESM.pdf]

Supplementary Material
Machine learning approaches for large scale classification of produce

Otkrist Gupta[1,*], Anshuman J. Das[1], Joshua Hellerstein[1], and Ramesh Raskar[1]

[1]Massachusetts Institute of Technology
[*]otkrist@mit.edu

1 Confusion matrix for apples (fine grained)

Here we show a sample confusion matrix obtained from taxonomy classification for apples.

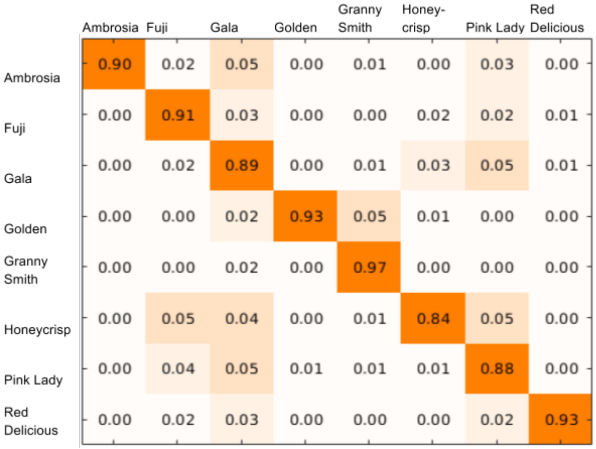

Figure 1: Confusion matrix for apple taxonomy identification. Eight different varieties are selected and analyzed.

2 Accuracy with variance for classification

Taxonomy	Instrument	Accuracy (± Var)	Cross Validation Accuracy (± Var)
Gala Apples	QEPro	96.5 (± 0.357)	95.7 (± 0.373)
	Flame NIR	65.2 (± 5.77)	62.3 (± 11.8)
	Flame	81.3 (± 14.9)	80.6 (± 9.87)
	NIRQuest	98.2 (± 0.512)	98.1 (± 0.322)
	Spark	95.3 (± 0.699)	94.3 (± 0.464)
Red Delicious Apples	QEPro	97.6 (± 0.823)	96.8 (± 0.456)
	Flame NIR	96.3 (± 0.651)	94.7 (± 0.959)
	Flame	91.5 (± 2.14)	91.3 (± 0.778)
	NIRQuest	97.6 (± 0.598)	97 (± 0.543)
	Spark	97.1 (± 0.863)	96.5 (± 0.453)
Navel Oranges	QEPro	97.1 (± 1.15)	96.6 (± 0.861)
	Flame NIR	59.4 (± 14.6)	71.6 (± 5.7)
	Flame	92.6 (± 2.13)	92.4 (± 0.815)
	NIRQuest	98.7 (± 1.07)	97.8 (± 1.22)
	Spark	96.7 (± 1.4)	96.3 (± 1.03)
Green Onions	QEPro	98.7 (± 1.05)	97.8 (± 0.741)
	Flame NIR	92.8 (± 3.07)	89.2 (± 2.53)
	Flame	94.3 (± 3.07)	92.8 (± 1.55)
	NIRQuest	98.8 (± 0.885)	97.7 (± 0.491)
	Spark	97.8 (± 1.41)	96.5 (± 1.51)
Bell Peppers	QEPro	96.1 (± 4.19)	95.5 (± 1.55)
	Flame NIR	96.2 (± 3.5)	94.7 (± 1.9)
	Flame	95.7 (± 2.72)	95.9 (± 1.44)
	NIRQuest	94.8 (± 2.71)	95.4 (± 1.66)
	Spark	98.9 (± 2.04)	99 (± 0.965)

Table 1: Classification accuracy for organic/inorganic classification with each instrument.

Instrument Name	Minimum (nm)	Maximum (nm)	Feature vector length
QEPro	406	780	1005
Flame NIR	1038	1542	89
Flame	347	1013	2009
NIRQuest	947	2075	473
Spark	363	697	985

Table 2: Feature vector length and span from each instrument (after clipping). We divided data in three separate files for organic/inorganic, taxonomy and farmer classification. We used pca based dimensionality reduction to compress observations and include coefficients from first 100 principal components as feature vectors for further analysis. These feature vectors can be downloaded from this link.

Taxonomy	Instrument	Accuracy (± Var)	Cross Validation Accuracy (± Var)
Fuji Apples	QEPro	99.3 (± 0.392)	98.8 (± 0.177)
	Flame NIR	80.7 (± 0.759)	79.8 (± 1.1)
	Flame	94.5 (± 1.12)	92.3 (± 1.77)
	NIRQuest	97.8 (± 0.713)	97.9 (± 0.45)
	Spark	95.5 (± 0.779)	94.9 (± 0.571)
Gala Apples	QEPro	99.5 (± 0.471)	99 (± 0.314)
	Flame NIR	95.5 (± 1.34)	95 (± 0.831)
	Flame	95.1 (± 1.44)	90.6 (± 3.75)
	NIRQuest	99.4 (± 0.506)	99.3 (± 0.38)
	Spark	98.4 (± 1.06)	98 (± 0.599)
Halo Oranges	QEPro	99.6 (± 0.35)	99.6 (± 0.108)
	Flame NIR	99.7 (± 0.3)	99.7 (± 0.168)
	Flame	97.4 (± 1.27)	96.5 (± 0.651)
	NIRQuest	100 (± 0.137)	99.8 (± 0.0847)
	Spark	99.6 (± 0.503)	99.3 (± 0.379)
Red Bell Peppers	QEPro	98.9 (± 0.921)	99 (± 0.31)
	Flame NIR	98.9 (± 0.842)	98.6 (± 0.544)
	Flame	93.8 (± 2.73)	93.1 (± 1.69)
	NIRQuest	99.1 (± 1.5)	99.3 (± 0.324)
	Spark	99.5 (± 0.623)	99.1 (± 0.396)
Red Potatoes	QEPro	99.3 (± 0.488)	99.1 (± 0.282)
	Flame NIR	94.9 (± 5.45)	94.1 (± 4.44)
	Flame	98.6 (± 0.889)	98.1 (± 0.482)
	NIRQuest	99.4 (± 0.307)	99.4 (± 0.292)
	Spark	99.3 (± 0.232)	99.4 (± 0.202)
Russet Potatoes	QEPro	99.9 (± 0.153)	99.8 (± 0.16)
	Flame NIR	85.6 (± 13.4)	81.5 (± 14.1)
	Flame	99.5 (± 0.348)	99.3 (± 0.273)
	NIRQuest	99.8 (± 0.27)	99.6 (± 0.147)
	Spark	99.9 (± 0.15)	99.8 (± 0.142)
Steak Tomatoes	QEPro	98.5 (± 0.798)	97.8 (± 0.599)
	Flame NIR	95.6 (± 1.29)	95.7 (± 0.994)
	Flame	85.7 (± 11.7)	90.1 (± 2.83)
	NIRQuest	99.1 (± 0.454)	99.2 (± 0.432)
	Spark	98.2 (± 1.12)	98 (± 0.427)

Table 3: Classification accuracy with instrument for farmer classification.

Taxonomy	Instrument	Accuracy (± Var)	Cross Validation Accuracy (± Var)
Apples	QEPro	90.8 (± 0.486)	90.2 (± 0.24)
	Flame NIR	51 (± 2.62)	54.1 (± 2.29)
	Flame	78.3 (± 2.16)	76.9 (± 1.68)
	NIRQuest	91.2 (± 0.405)	90.3 (± 0.201)
	Spark	89.9 (± 0.461)	88.7 (± 0.195)
Strawberries	QEPro	86.7 (± 1.86)	83.4 (± 1.13)
	Flame NIR	74.5 (± 3.45)	75.4 (± 2.22)
	Flame	84.6 (± 1.85)	83.3 (± 1.84)
	NIRQuest	92.9 (± 1.62)	91.6 (± 0.876)
	Spark	87.8 (± 2.52)	87.1 (± 0.857)
Grapes	QEPro	95.6 (± 1.11)	95.6 (± 0.4)
	Flame NIR	24.6 (± 4.79)	75.6 (± 7.9)
	Flame	88.4 (± 1.5)	87.7 (± 1.66)
	NIRQuest	91.1 (± 1.41)	90 (± 1.08)
	Spark	95.6 (± 0.716)	95.4 (± 1.03)
Oranges	QEPro	98.5 (± 0.336)	97.9 (± 0.311)
	Flame NIR	90 (± 1.04)	89 (± 0.885)
	Flame	91.9 (± 0.907)	91.4 (± 0.553)
	NIRQuest	98.1 (± 0.425)	97.7 (± 0.203)
	Spark	98 (± 0.361)	97.5 (± 0.325)
Mushrooms	QEPro	96.5 (± 0.878)	95.6 (± 0.672)
	Flame NIR	84.6 (± 2.68)	83.4 (± 1.11)
	Flame	85.9 (± 1.25)	82.8 (± 0.726)
	NIRQuest	93.8 (± 1.42)	92.7 (± 0.406)
	Spark	94.9 (± 0.886)	93.9 (± 0.848)
Onions	QEPro	97.4 (± 0.493)	96.7 (± 0.412)
	Flame NIR	64.6 (± 1.33)	63.3 (± 1.37)
	Flame	92.6 (± 0.983)	91.8 (± 0.389)
	NIRQuest	90.7 (± 0.942)	88.6 (± 0.55)
	Spark	96.9 (± 0.989)	97 (± 0.351)
Bell Peppers	QEPro	96.9 (± 0.522)	96.3 (± 0.381)
	Flame NIR	83.5 (± 1.36)	81.8 (± 0.754)
	Flame	88.2 (± 1.91)	87.9 (± 0.985)
	NIRQuest	95 (± 0.688)	93.9 (± 0.762)
	Spark	97.1 (± 0.697)	97.3 (± 0.302)

Table 4: Accuracy by instrument for taxonomic identification.

3 Analysis of model selection

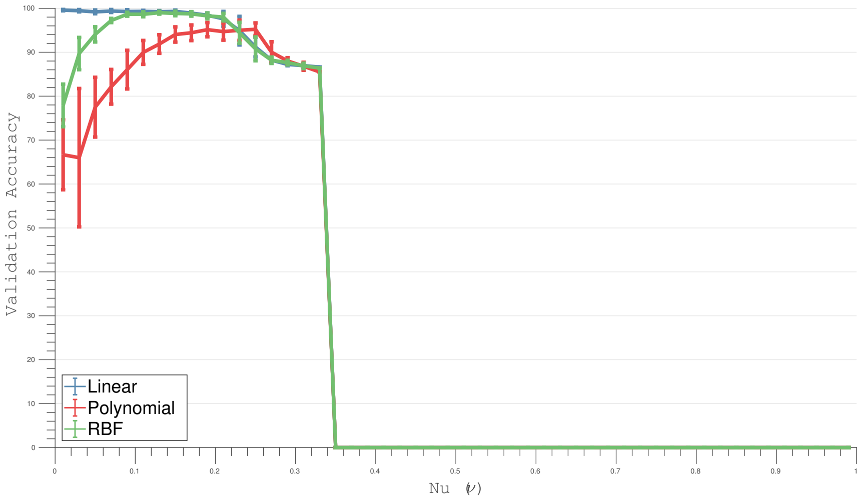

(a) Model selection for farmer classification over halo oranges.

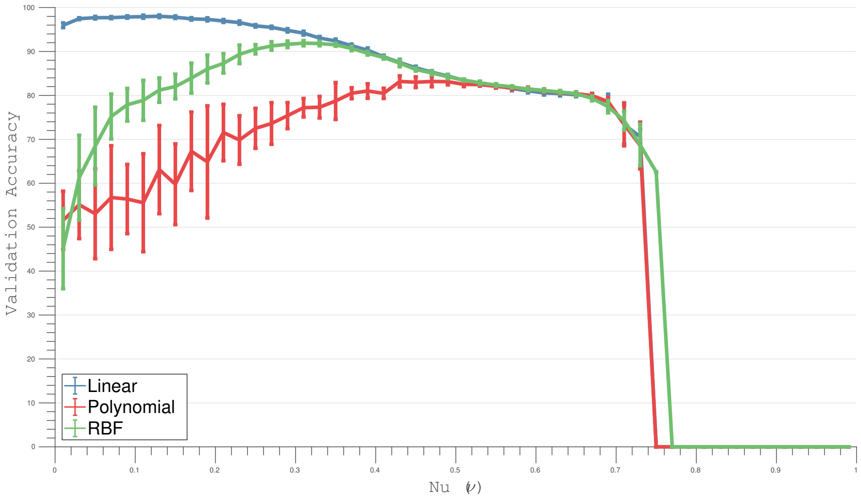

(b) Model selection for organic classification of gala apples.

Figure 2: Model selection with hyperparameter ν in SVM implementation. Accuracy can greatly vary by hyperparameters including ν and t. We perform a grid search to search for best performing value of both ν and t.

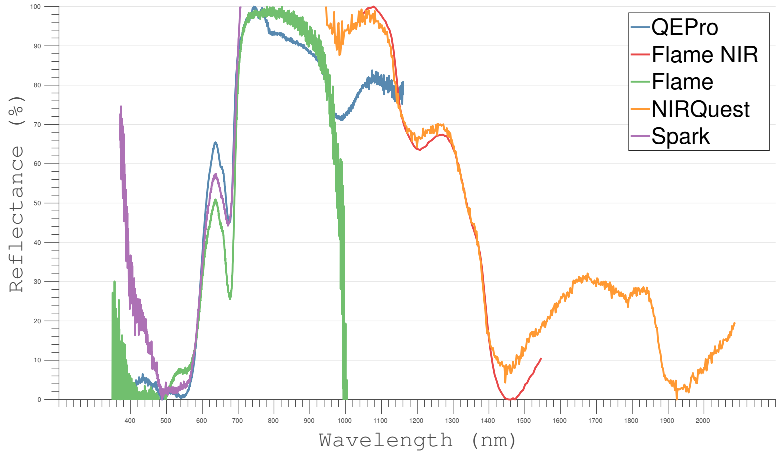

Figure 3: Recorded measurements from multiple instruments for a single Fuji apple sample. Different colors represent measurements from different recording instruments. While instruments agree on most variations, there are differences originating from calibration error in each instrument.

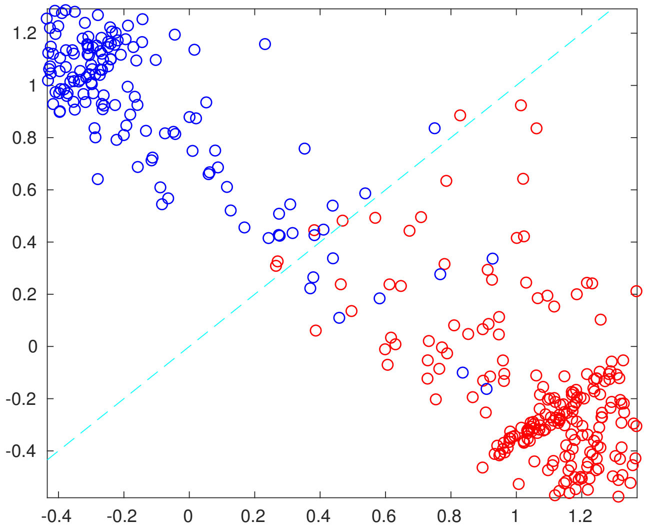

Figure 4: Separating hyperplane for organic and inorganic classification for Fuji apples. Red marks organic samples, blue marks inorganic samples, and the dashed line represents the separating hyperplane.
